# Decoding Spatial Complexity of Diverse RNA Species in Archival Tissues

**DOI:** 10.1093/gpbjnl/qzae089

**Published:** 2024-12-18

**Authors:** Junjie Zhu, Fangqing Zhao

**Affiliations:** Institute of Zoology, Chinese Academy of Sciences, Beijing 100101, China; Key Laboratory of Systems Biology, Hangzhou Institute for Advanced Study, University of Chinese Academy of Sciences, Hangzhou 310024, China; Institute of Zoology, Chinese Academy of Sciences, Beijing 100101, China; Key Laboratory of Systems Biology, Hangzhou Institute for Advanced Study, University of Chinese Academy of Sciences, Hangzhou 310024, China; University of Chinese Academy of Sciences, Beijing 100049, China

## Background

Spatial transcriptomics has recently emerged as a powerful tool for deciphering the spatial organization of gene expression *in situ*, providing critical insights into tissue architecture, disease progression, and cellular interactions [1–3]. Notably, microfluidic-based spatial transcriptomics technologies provide distinguished cost-effectiveness, flexible designs, and widespread accessibility within the community [4,5]. Formalin-fixed paraffin-embedded (FFPE) tissues, routinely used in histopathology for clinical diagnosis, are a valuable resource for human biology research, with extensive repositories accumulated over decades [6,7]. However, RNA in FFPE tissues frequently undergoes fragmentation, degradation, or chemical modifications during embedding process and storage [8], which has historically hindered their utility in conventional poly(A)-based spatial transcriptomic studies. Moreover, most spatial transcriptomics technologies primarily focus on messenger RNA (mRNA), with limited capacity to map the full landscape of diverse RNA species and their functional dynamics [9,10].

**Figure 1:**
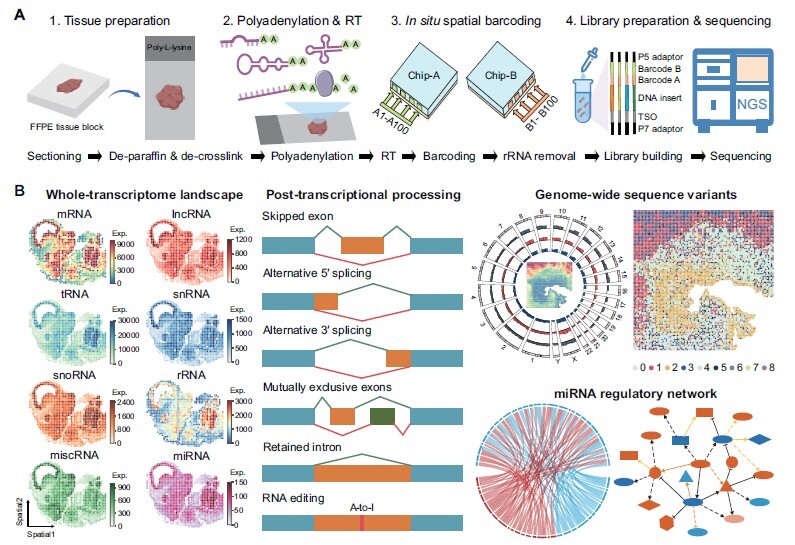
The workflow and applications of Patho-DBiT **A**. Overview of the Patho-DBiT workflow. The FFPE tissue blocks were sectioned and mounted onto the center of a poly-L-lysine coated glass slide. The sections then underwent paraffin removal and de-crosslinking before the intact tissue was imaged. After tissue permeabilization, RNA was polyadenylated and reverse transcribed to cDNA. Then two rounds of orthogonal PDMS chips were used to spatially barcode the cDNA in tissue. After cDNA extraction and amplification, rRNA components were removed and the resultant products were subjected to the following library preparation and sequencing steps. **B**. Downstream data analysis enabled by Patho-DBiT, including spatial profiling of the whole transcriptome, post-transcriptional processing (*e.g*., alternative splicing and RNA editing), genome-wide spatial sequence variant profiling, and identification of miRNA-associated regulatory networks. FFPE, formalin-fixed paraffin-embedded; RT, reverse transcription; cDNA, complementary DNA; PDMS, polydimethylsiloxane; TSO, Template switch oligo; NGS, next-generation sequencing; rRNA, ribosomal RNA; mRNA, messenger RNA; tRNA, transfer RNA; snoRNA, small nucleolar RNA; miscRNA, miscellaneous RNA; lncRNA, long non-coding RNA; snRNA, small nuclear RNA; miRNA, microRNA; Exp., expression; A-to-I, adenosine-to-inosine.

To address these limitations, Bai et al. [11] developed Patho-DBiT, a cutting-edge spatial transcriptomics platform specifically tailored to unravel the complex RNA biology in clinically archived FFPE tissues (**Figure 1**A). This innovative method integrates *in situ* polyadenylation, microfluidic tissue barcoding, and advanced computational approaches. By appending poly(A) tails, Patho-DBiT enables genome-wide spatial co-profiling of mRNAs, large and small non-coding RNAs (ncRNAs), alternative splicing, and sequence variants, providing exceptional coverage and sensitivity. The authors further demonstrated the platform’s ability to dissect the spatiotemporal complexity of clinical samples.

## Expanded RNA profiling capabilities

Patho-DBiT demonstrates superior capabilities in mapping the spatial distribution of diverse RNA species at the whole-transcriptome level, ensuring enhanced detection across various tissue types and resolutions (Figure 1B). By incorporating *in situ* polyadenylation and ribosomal RNA (rRNA) depletion, this platform achieves a 10- to 100-fold increase in ncRNA capture compared to the previous DBiT-seq protocol [5], while maintaining high sensitivity for mRNA detection. Particularly, it shows notable precision and sensitivity in detecting small RNAs, such as microRNAs (miRNAs). Profiling of mouse brain sections further validated Patho-DBiT’s ability to concurrently assess gene expression and post-transcriptional modifications, covering all major splicing events and adenosine-to-inosine (A-to-I) RNA editing, with results corroborated by long-read sequencing data. The analysis unveiled region-specific isoform expression and distinct A-to-I editing patterns across different brain regions, correlating these molecular events with respective brain functions.

## Insights into tumor heterogeneity and progression

Even when applied to angioimmunoblastic T-cell lymphoma samples stored for over five years, Patho-DBiT effectively revealed the spatial gene expression landscape and the signaling pathways driving lymphomagenesis, as validated by high-plex immunofluorescence using CODEX technology. Similarly, in extranodal marginal zone lymphoma of mucosa-associated lymphoid tissue (MALT) samples stored for three years, the platform accurately identified cellular clusters corresponding to histological structures, even detecting rare cell populations in specific regions. By integrating histological images for super-resolution mapping, the authors achieved single-cell spatial resolution across the entire tissue, significantly enhancing the understanding of cellular heterogeneity.

The authors delved deeper into the tumor discrimination, progression, and associated regulatory networks. They resolved the developmental trajectory and putative driver genes in malignant B-cell progression within the MALT sample, aided by high proportion of intronic reads. Through single nucleotide variation (SNV) profiles generated from genome-wide sequence variants, the platform distinguished malignant from non-malignant cells and traced subclonal evolution at different stages, independent of known markers. Additionally, they conducted a comprehensive spatial analysis of 1352 detected miRNAs, spatially mapping regulatory networks involved in tumorigenesis, such as miR-21 and miR-155. Finally, the authors devised single-cell spatial analysis of diffuse large B-cell lymphoma sections from a different gastric region of the same patient alongside the MALT sample. The precise cellular-level molecular mapping of the tumor progression from low-grade to high-grade tumors shed light on the activation of NF-κB associated pathways and inhibition of tumor suppression pathways, and revealed the complex tumor microenvironment, such as B-cell clonality and macrophage-tumor interaction, in the context of aggressive lymphomas.

## Discussion

This study represents a significant advancement in spatial transcriptomics, providing a robust and sensitive platform for transforming spatial transcriptomic information in archival FFPE tissues. By enabling spatial co-profiling of diverse RNA species, post-transcriptional processing, and genome-wide SNVs, Patho-DBiT opens new avenues for investigating tumor heterogeneity, progression, and the tumor microenvironment in clinical samples. One of its most notable achievements is the enhanced ability to profile small ncRNAs, such as miRNAs, which even outperforms its application in frozen samples. This observation suggests that different RNA species may undergo varying degrees of degradation in FFPE tissues, with miRNAs potentially preserved due to the protecting effects of RNA-binding proteins. These findings highlight the necessity of accounting for factors that influence RNA quality and quantification in archived samples, thereby minimizing the risk of erroneous interpretations.

Patho-DBiT also demonstrates exceptional promise in elucidating cancer structural heterogeneity and its progression. By enabling spatially resolved analyses of distinct cancer stages and tumor microenvironments, the platform provides critical insights into cellular composition and gene expression variations within tumors. Its ability to map the spatial distribution of gene expression, post-transcriptional modifications, and sequence variants facilitates the identification of early biomarkers and novel therapeutic targets. Furthermore, Patho-DBiT could offer valuable perspectives on tumor progression, exemplified by its application in mapping gene expression across diverse cancer cell types in aggressive lymphoma samples. While the current platform lacks single-cell resolution and may exhibit limited coverage for certain alternative splicing events, its transformative potential in spatial oncology remains unequivocal.

Looking forward, Patho-DBiT is poised to revolutionize our understanding and diagnosis of diseases through retrospective studies of long-preserved clinical specimens. These archival tissues, accumulated over decades, represent an unparalleled resource and harness a wealth of information for biomedical research. Integrating spatial multi-omics with traditional pathological methods, Patho-DBiT has the potential to bridge the gap between classical pathology and modern molecular analysis, equipping pathologists and researchers with advanced tools to dissect the molecular basis of diseases, ultimately paving the way for improved diagnostic and therapeutic strategies.

## CRediT author statement

**Junjie Zhu:** Investigation, Writing – original draft. **Fangqing Zhao:** Conceptualization, Investigation, Supervision, Writing – review & editing. Both authors have read and approved the final manuscript.

## Competing interests

Both authors have declared no competing interests.
